# Nano-Aptasensing in Mycotoxin Analysis: Recent Updates and Progress

**DOI:** 10.3390/toxins9110349

**Published:** 2017-10-28

**Authors:** Amina Rhouati, Gonca Bulbul, Usman Latif, Akhtar Hayat, Zhan-Hong Li, Jean Louis Marty

**Affiliations:** 1 Ecole Nationale Supérieure de Biotechnologie, Constantine 25100, Algerie; amina.rhouati@gmail.com; 2 BAE: Biocapteurs-Analyses-Environnement, Universite de Perpignan Via Domitia, 52 Avenue Paul Alduy, 66860 Perpignan CEDEX, France; 3 Department of Chemistry and Biomolecular Science, Clarkson University, Potsdam, NY 13699-5810, USA; bulbulg@clarkson.edu; 4 Interdisciplinary Research Centre in Biomedical Materials (IRCBM), COMSATS Institute of Information Technology, Lahore 54000, Pakistan; usmanlatif@ciitlahore.edu.pk (U.L.); akhtarhayat@ciitlahore.edu.pk (A.H.); 5 School of Environmental and Materials Engineering, College of Engineering, Shanghai Polytechnic University, Shanghai 201209, China; zhli@sspu.edu.cn

**Keywords:** nanomaterials, gold/silver nanoparticles, metal oxides, aptamer, mycotoxins, food analysis

## Abstract

Recent years have witnessed an overwhelming integration of nanomaterials in the fabrication of biosensors. Nanomaterials have been incorporated with the objective to achieve better analytical figures of merit in terms of limit of detection, linear range, assays stability, low production cost, etc. Nanomaterials can act as immobilization support, signal amplifier, mediator and artificial enzyme label in the construction of aptasensors. We aim in this work to review the recent progress in mycotoxin analysis. This review emphasizes on the function of the different nanomaterials in aptasensors architecture. We subsequently relate their features to the analytical performance of the given aptasensor towards mycotoxins monitoring. In the same context, a critically analysis and level of success for each nano-aptasensing design will be discussed. Finally, current challenges in nano-aptasensing design for mycotoxin analysis will be highlighted.

## 1. Introduction

The word Mycotoxin is derived from two Greek words “mikes” and “toxin”, meaning “fungi” and “poison”, respectively [1]. Thus, Mycotoxins are poisonous compounds that are produced as secondary metabolites by certain fungi, being present in food and feed. These toxic chemical compounds (MW ~ 700) can be produced during harvesting, storage, processing and distribution of crops [2]. These mycotoxins contaminate about 25% of world’s food production, based on the report of United Nations of Food and Agriculture Organization. These toxins either destroy million tons of food each year or divert it into a form which is unsuitable for human consumption [3]. More than 300 mycotoxins have been discovered so far. The consumption of these toxins causes severe effects on animal and human health [4,5]. Researchers have mainly focused on those toxins which are proven to be carcinogenic or toxic such as aflatoxins (AFs), ochratoxins (OT), fumonisins (Fs), zearalenone (ZEN), patulin (PAT), tremorgenic toxins trichothecenes, and ergot alkaloids [4].

The WHO-International Agency of Research on Cancer has classified these toxins and put AFs in Group 1, OT and Fs in Group 2B, and trichothecenes and ZEN in Group 3. In the same context, AFs is in the category of carcinogenic, OT and Fs as possibly carcinogenic, and trichothecenes and ZEN are non-carcinogenic for human [6]. It is necessary and crucial to analyze mycotoxins to ensure food safety because of serious health risks associated with mycotoxins and its effect on domestic and international trade. There are a number of conventional analytical techniques available for mycotoxin detection. 

The conventional methods to detect/quantify mycotoxins include the chromatographic separation and then its detection via a suitable detector. In these methods, mycotoxins in food and feed can be determined by separating with High Performance Liquid Chromatography (HPLC) or Gas Chromatography (GC) and then subsequent detection with fluorescence detector, ultraviolet detector or using a mass spectrometer [7,8,9]. Standardized reference analytical methods for mycotoxins (aflatoxins, ochratoxins, fumonisins, PAT, and DON) detection in food have been made available from different official authorities, such as the European Committee for Standardization (CEN), and international organization for standardization [10,11]. Thin-layer chromatography (TLC) is a simple and low-cost qualitative method for mycotoxins analysis, but cannot be used for sensitive or precise measurements [12]. Gas chromatography (GC) coupled with flame ionization detector (FID) or mass spectrometry (MS) is the most widely used method for sensitive detection of trichothecenes [9]. A diode array detector, UV or fluorescence detector coupled with HPLC is used for the detection of aflatoxins, ochratoxin A, or citrinin in food. LC coupled with mass spectrometry or tandem mass spectrometry (LC-MS/MS) is the most promising method for simultaneous detection of number of mycotoxins [13,14,15,16,17,18]. 

Apart from chromatographic based methods, there are different optical methods to analyze mycotoxins in food. Optical waveguide light spectroscopy (OWLS) was utilized to measure OTA and AFB_1_ in food samples but this technique was not sensitive enough [19]. Near-infrared spectroscopy (NIR) is faster and better than destructive techniques for analyzing mycotoxins in cereals [20]. There are different modes of NIR spectroscopy such as transmittance, diffuse transmittance, diffuse reflectance, and transflectance. Diffuse transmittance is suitable for detecting mycotoxins in 1–2 cm thick samples such as meat and grain, whereas diffuse reflectance is useful for thicker samples. Mid-infrared spectroscopy with attenuated total reflection (MIR-ATR) is also a suitable non-invasive technique for monitoring mycotoxins in grains [21]. Evanescent wave technology was used to develop fiber-optic immunosensor for FB_1_ and AFB_1_ in maize by anchoring monoclonal antibodies. Scientists have also explored surface plasmon resonance (SPR) technology for detecting DON, ZEA, AFB_1_, OTA and FB_1_ with good detection limit and time frame [22]. Other optical methods to monitor mycotoxins include circular dichroism [23], optical rotatory dispersion [24]. Most of the above-mentioned techniques are very accurate but certain limitations are associated with these techniques such as the high cost, and the need for bulky instruments and skilled persons. In this direction, biosensors are very accurate, simple and cost-effective alternatives for a quick on-spot analysis of mycotoxins. A bio-recognition element, specific to target analyte, is anchored on the transducer surface to fabricate a biosensor. The most common bio-receptor molecules used in biosensor construction include enzymes, antibodies and aptamers [2,25]. However, there are various drawbacks while using enzymes in biosensors assay field such as: (1) enzyme denaturation induced by change in temperature, pH, pressure, radiation, and certain chemicals; (2) change in sensing enzymatic reaction by conformational change in amino acids located at active sites; and (3) loss in enzymatic activity during the enzyme transfer after isolation to an in vitro operating temperature. Thus, specific working conditions are required for stable sensing enzymatic activity and scientists are also working to improve the enzymatic stability [26]. Immunoassays are an alternative to enzymatic assays which are based on antigens and antibodies interactions [27]. These assays offer higher sensitivity but poor LOD and are also prone to denaturation because antibodies are proteinic in nature. These antibodies are very costly because they are produced in living animals. Moreover, optimum physiological conditions are required for proper sensing ability of antibodies. 

Alternatively, a new class of molecules called aptamers are introduced which are promising diagnostic tools for different analytes. These aptamers are composed of single stranded oligonucleotides (DNA or RNA) and interact with analytes with antibody-like ability. These aptamers are folded in a well-defined three-dimensional structures and have significant advantages over antibodies [28,29]. Aptamers are cheaper than antibodies because they can be prepared chemically without the need for animal immunization. Another advantage of aptamers is their easy labeling with fluorescent dyes, enzymes, biotin, etc. making them suitable for a variety of detection methods [30,31,32,33,34,35,36]. The aptamers can be easily regenerated and reused for other analytes. All of the above-mentioned advantages make aptamers, in comparison to antibodies as well as other bio-receptors, promising candidates for fabricating analytical devices for various analytes. Similarly, the increasing trend in the domain of nanotechnology with the successful synthesis and characterization of a variety of nanomaterials has opened a new era to design transducer surfaces with unique optical, electronic, magnetic and catalytic properties [37,38]. Nanomaterials have found wider applications in the field of energy harvesting [39] and information technology [40], and researchers have synthesized nanomaterials that are very well integrated in the fabrication of biosensor [41]. Thus, the combination of nanomaterials with aptamers provides a potentially promising design of aptasensing platforms. The next section is focused on the type of nanomaterials that have potential for integration into the fabrication of aptamer assays for mycotoxin analysis. 

## 2. Integration of Nanomaterials in Aptasensors

The use of nanomaterials in the biosensing has been increasing tremendously in the last decade. Enhanced performances with increased selectivity and sensitivity are the main outcomes of nanoparticle integration [42]. Additionally, nanoparticles present a higher surface area, enabling the immobilization of increased amount of biosensing elements such as antibodies, enzymes, receptors and aptamers [43]. The presence of well-developed synthesis methods for nanomaterials (e.g., noble metal, magnetic, and metal oxide nanoparticles) offers a wide range of selection options for the right employment of the sensing element in the aptasensing design. Due to their small size (1–100 nm), these nanoparticles possess unique optical, chemical, and electronic properties that differs from their bulk materials, promoting their use in the aptasensors [44]. The majority of the nano-aptasensors developed up to date are based on noble metal nanoparticles due to their unique physicochemical properties [45]. Within the noble metal nanoparticles group, gold nanoparticles have been employed more in sensing owing to their biocompatibility and optical properties [46]. Nano-silver has also found applications in aptasensing for detection of a broad range of molecules. Carbon based nanomaterials, magnetic and metal oxide nanoparticles are also applied in aptasensing and will be discussed in detail in the following sections with examples of their applications in recent literature.

### 2.1. Types of Nanomaterials

#### 2.1.1. Gold Nanoparticles

Numerous detection schemes of aptasensing have been drawn by investigating different physicochemical properties of gold nanoparticles, such as fluorescence quenching [47], electrochemical activity [48], surface-enhanced Raman scattering (SERS) [49], and localized surface Plasmon resonance (LSPR) [50]. In between these schemes, colorimetric detection has become the most studied and employed method due to its simplicity and increased opportunities in employing on portable platforms. Because of the collective oscillation of free electrons on their conductive bands, gold NPs possess intense surface plasmon absorption bands [51], facilitating the easy adoption of these particles in colorimetric aptasensors. Additionally, they exhibit size and distance dependent optical properties which is of great interest in colorimetric aptasensor design [52]. Gold nanoparticles change color from red to purple/blue during aggregation or purple/blue to red during redispersion of gold nanoparticle aggregates [53]. This behavior is due to the changes in the interparticle plasmon coupling resulting in changes in the surface plasmon band shift. Therefore, the assembly/disassembly of nanoparticles and resulting changes in the color have been extensively employed as an indicator in colorimetric assays for many molecules that induces aggregation/redispersion. 

Gold nanoparticles provide an appropriate environment for biomolecule immobilization and facilitates the electron transfer between immobilized sensing probes and electrode surfaces, which resulted in intensive use of gold nanoparticles for development of electrochemical aptasensors with enhanced analytical performance [54,55]. Electrochemical aptasensors track the electrochemical changes that occur when the sensing surface of the detecting electrode interact with chemicals of interest. They can be divided into different categories based on their sensitivity to changes in the detectable signals: (1) potentiometric, measures the changes in the voltage between the electrodes; (2) amperometric, measures the changes in the current at a given applied voltage; and (3) conductometric, measures changes in the ability of the sensing material to transport charge (electron). Gold nanoparticles have been already employed in constructing electrochemical sensors based on all above-mentioned signals. The main reason for using gold nanoparticles in electrochemical aptasensors is to obtain higher sensitivity based on their electrocatalytic and surface properties.

The use of gold nanoparticles is also exploited for Förster resonance energy transfer (FRET)-based aptasensors. Gold nanoparticle based fluorescence aptasensors have been employed to detect mycotoxins such as Ochratoxin A [56], and Aflatoxins [57] based on different mechanisms. Mainly, nanoparticle has been employed to mediate the quenching of fluorescent dyes, increasing the sensitivity and efficiency of the assays. The following are the two main approaches that have been utilized: (A) molecular beacons; and (B) hybrid structures with DNA. In the following section, we have given the detailed explanation on the mechanisms for each.

#### 2.1.2. Carbon Based Nanoparticles

Fullerenes, single- or multi-walled carbon nanotubes, and graphene are called carbon-based nanomaterials [58]. In the last decade, researchers have applied them in various aptasensing designs with electrochemical, colorimetric, fluorimetric outcomes for detection of different analytes. Graphene is mostly employed in electrochemical aptasensor designs, whereas nanotubes are mostly assayed by monitoring fluorescence response. Extraordinary electronic transport properties of individual graphene sheets have been demonstrated in several areas since the exfoliation and characterization of graphene have shown in 2004 [59,60]. After that, it has found widespread application in electrochemical analysis owing to their unique conductivity [61] and has gained tremendous interest both in academics and industry. It has been shown that carbon nanotubes can act as quenchers for different fluorophores by electron transfer process, which gives low background with high signal-to-noise ratio [62]. Fluorescence quenching is led between fluorophore labeled aptamer and nanotubes because of the π–π stacking interaction between the DNA bases of the aptamer and carbon nanotubes once they are close to each other [63]. Later on, the signal can be recovered by addition of the target molecule, or the complementary strand of the ssDNA. 

#### 2.1.3. Metal Oxide Nanoparticles 

The presence of a broad range of electronic, physical and chemical characteristics make metal oxides, especially doped structures, very sensitive to the changes in their environment [64]. Previously, these interesting properties of metal oxides, e.g., SnO_2_, In_2_O_3_, ZnO, and TiO_2_, have been extensively utilized for development of gas sensors [65]. Later on, they have attracted researchers interests as an effective surface in biosensor design for biomolecule immobilization with better conformation and higher sensing characteristics [66]. These particles are easy to modify enabling tailoring of size, surface area, functionality, adsorption capacity and electron-transfer properties. TiO_2_ [67], CeO_2_ [68], and Fe_3_O_4_ [69] have been used in conjunction with different bioreceptors: enzymes, antibodies, and recently DNA based on several detection mechanisms. 

#### 2.1.4. Quantum Dots (QDs)

Known as zero dimensional particles, QDs are bright NPs which have been applied frequently as fluorescent probes [70] due to unique photophysical properties such as wide absorption spectra and narrow photoluminescence spectrum. It was shown that QDs present 10–20 times more brilliant fluorescence and their photodurability is 100 times better than organic dyes [71], which make them one popular choice in designing fluorescence based aptasensors. QDs are often used as FRET donors for organic dye acceptors [72] and their emission can be size-tuned [73]. Additionally, it is possible to excite different-colored QDs by a single light source, and they will still produce specific, narrow and symmetric emissions of different colors, which can be point of interest for multiplexing and array development [38].

### 2.2. Functions of Nanomaterials in Aptasensors

While a huge number of nanoparticle-based assays have been developed and reported for aptasensor construction, we have classified these approaches based on the specific function of nanomaterials in a given assay.

#### 2.2.1. Immobilization Support

**Figure 1:**
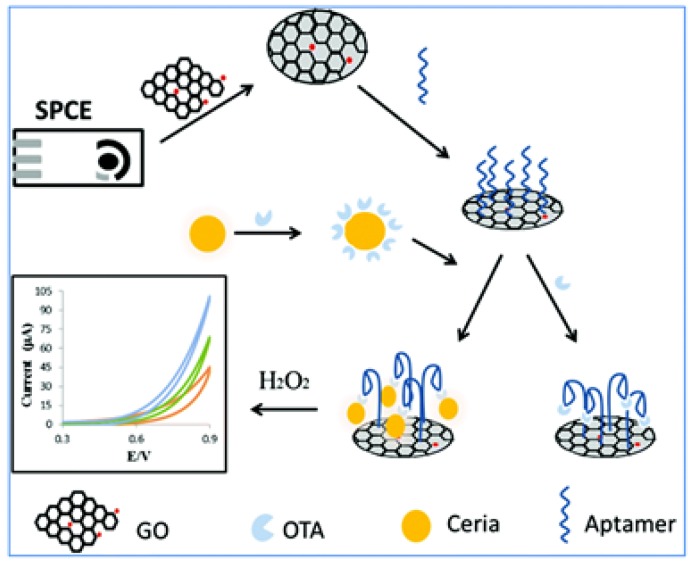
Schematic illustration of the non-enzymatic nanocatalyst based electrochemical aptasensor concept involving the use of a nCe tag and GO for Ochratoxin A detection corn sample, reproduced with permission from [67]. Copyright Royal Society of Chemistry, 2015.

In aptasensor applications, indicator molecules (e.g., dyes, nanoparticles, and reagents) are usually employed in immobilized form. Previously, polymers, glasses, sol-gels, hydrophilic molecules such as polysaccharides and hydrophobic materials such as polyvinylchloride were used as supports in aptasensor design [74]. Later, nanomaterials found application as immobilization support due to their various characteristics, such as higher and selective permeability capacities for certain molecules. The use of NPs in the design of aptasensors brings the following opportunities: (1) easier application towards commercialization due to the possible increase in physiological stability; (2) reducing the diffusion limits; (3) maximizing the surface area, and, consequently, increasing the biomolecule loading; and (4) easier modification, which enables producing charged NPs that can be used in attaching biomolecules with different charges. Various nanomaterials have been synthesized in different structures such as nanorods, nanowires, nanotubes for their adoption as immobilization platforms [75]. Their tendency to adsorb biomolecules makes these structures appealing choice for their application in aptasensor construction. The adsorption of aptamers into the bulk materials as immobilization support may result in loss of bioactivity, and their distribution on the platform may not be even. On the other hand, integration of nano-size materials helps retain the bioactivity of the aptamers while enabling even distribution of these biomolecules [76]. To enable the attachment of the aptamers on the particle surface, it is essential to functionalize NPs to carry different charges. Therefore, it would be possible to link the aptamers with different charges to NPs by means of electrostatic interactions while contributing the development of layer by layer assemblies. Additionally, affinity based methods by employing avidin/biotin or streptavidin/biotin complexes and immobilization by covalent bonding are other techniques utilized in aptasensor construction [77,78]. While the presence of various chemical and physical properties at the same time on the same NP looks appealing, it can bear some problems for their particular application. For instance, the presence of intrinsic enzyme-like properties can allow the development of enzyme-free sensors to detect target molecules. However, the same property can restrict their application as immobilization support. Consequently, it is crucial to perform control experiments with these NPs before applying them as immobilization support in aptasensors. Due to their reactivity with commonly used signal-generating probes, they can produce irreproducible data. Surfactants, encapsulation techniques, variations in the parameter such as pH can be employed to control the unspecific surface reactivity and adsorption. Bulbul et al. used redox active nanoceria particles as both catalytic label and redox mediator and reduced graphene oxide as immobilization platform in the same assay for sensitive and selective detection of Ochratoxin A [68] (Figure 1).

#### 2.2.2. Mediator

It is crucial to create an electrical contact between redox active biomolecules and the electrode surface while developing electrochemical aptasensors. On the other hand, in some specific cases the redox active moieties can be blocked by thick insulating proteins and/or different molecules hindering the electron transfer. In these conditions, to increase the electron transfer between the biomolecules and the transducer surface, NPs can be used. Metallic [79], oxide [80] and semiconductor [81] NPs have been previously employed to improve the electron transfer rate. Conductivity of NPs and their well-defined arrangements can enable development of nano-based methods for construction of sensitive aptasensors with enhanced electron transfer properties. For example, Sun et al. took advantage of synergistic contribution of chitosan–gold NPs, grapheme-gold NPs and multi-walled carbon nanotubes as the nanocomposites mediator and improved the electron replay during the electron transfer process [82]. In another recent study, Shi et al. developed an ultra-sensitive aptasensor by employing ionic and Fe_3_O_4_ as the composite mediators. Accelerated electron transfer with improved response speed and precision was obtained by depositing these materials onto a screen-printed carbon electrode [69]. However, the use of these NPs as mediators in aptasensing for mycotoxin detection is still in their infancy.

#### 2.2.3. Signal Amplification

Due to the need for ultrasensitive aptasensors with miniaturized platform, the integration of NPs as signal amplification attracted considerable attention. NPs can be employed as both electronic and optical tags to amplify the signal. Different characteristics of each NP can result in increase in the signal, such as ability to increase the conductivity, and/or intrinsic catalytic activity over the molecules which can be an interest for their integration in electrochemical aptasensors. In colorimetric sensing, ability to increase the signal brightness, photostability and multiplexing capabilities of particular NPs such as nanobeads and quantum dots enable the signal amplification. Signal amplification in aptasensing by using NPs is possible by following mechanisms: through catalytic reaction, by using them as mediator, and by employing them as mediators to deposit electrochemically active species. Catalytic properties of silver NP, carbon nanotube, graphene oxide and platinum NPs in electrochemical detection [83,84] for amplified detection of biorecognition events were previously described in aptasensing, and are examples of signal amplification through catalytic reaction. Palladium NPs were also used as catalyst for H_2_O_2_, and enabled the construction of a sensitive electrochemical aptasensor [85]. The integration of NPs with the aptamers is related to the improvement of aptasensor performance, and is a factor enabling their use as mediators. Gold NPs have also been utilized in colorimetric aptasensors to increase the signal due to their high extinction coefficients and strong distance-dependent optical properties [86]. Carbon-based nanomaterials are frequently applied as signal amplifiers in electrochemical aptasensing because they promote and facilitate the electron transfer between the biomolecules and the electrode surface [87]. Additionally, they interact with other nanomaterials such as gold NPs, SiO_2_, chitosan, etc., resulting in their widespread application in aptamer based electrochemical bioassays [88]. 

#### 2.2.4. Alternative to Enzyme Labels

Several nanozymes have been developed and reported which can mimic enzymes for their applications in the biosensing as labels. Carbon-based NPs such as graphene and carbon nanotubes; metal-based NPs such as Au, Ag, and Pt; and metal oxide-based NPs such as cerium oxide and Fe_2_O_4_ have been extensively studied [89]. The enormous interest that NPs received is due to their distinct properties such as high surface to volume ratio, abundance of reactive groups on their surfaces. Catalase, oxidase, peroxidase like activities of NPs have been shown for several types of nanomaterials [90]. Mostly, these nanozymes exhibit environment dependent properties such as changes in their activity at different pHs or with surface modifications. These tunable characteristics can result in changes in their affinities between nanozymes and their substrates, enabling development of novel aptasensors for many target analytes. Low cost, high stability and sustained catalytic activities are some of the advantages that they offer. On the other hand, it is difficult to regenerate NPs surface and control the reactivity of nanomaterials against certain interfering molecules. Therefore, studies on design and development of selective and specific NPs to overcome the matrix interferences are needed to replace enzymes for the aptasensing applications. 

#### 2.2.5. Optical Signal Generating Probe

Due to the intrinsic physicochemical properties that NPs possess, they have been considered as ideal signal generating probes [38]. Aggregation-induced interparticle surface plasmon coupling of gold NPs, which give a visible color change from red to blue, resulted in their extensive use in colorimetric sensing. This concept has been used as a practical platform for detection of any target analyte that causes gold NPs aggregation or re-dispersion [46]. The adsorption of single-stranded DNA on negatively charged gold NPs by electrostatic interactions and stabilization against salt induced aggregation was shown by Li and Rothberg [91]. They have shown the application of this phenomenon for a hybridization assay based on color changes associated with the gold aggregation. Another strategy was developed by F. Li et al. for colorimetric detection in which the aptamer was divided into two subunits and attached to different gold NPs. In the absence of target, particles were dispersed, whereas in the presence of target as linker, the subunits came together and lead to the aggregation of NPs with a change in the color of the solution [92]. Zhang et al. also divided the aptamer into two parts, and adsorbed it on the gold NP surface, which protected the salts induced aggregation. Then, after addition of the target, the folding occurred, and the particles were unprotected and resulted in the aggregation of gold NPs with color change [93]. 

#### 2.2.6. Fluorescence Quencher

**Figure 2:**
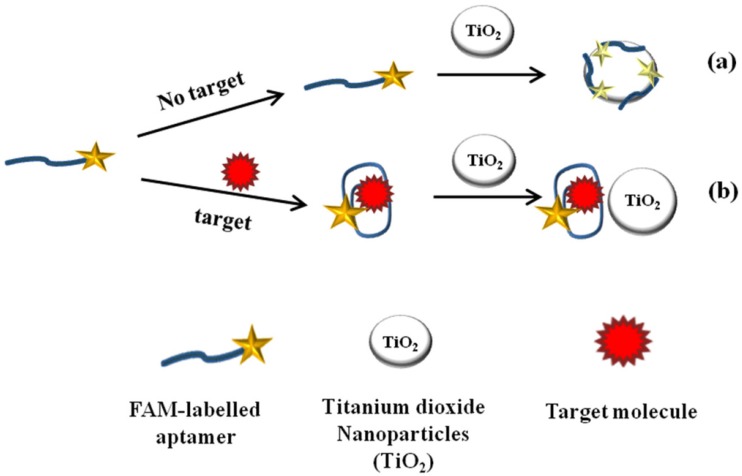
TiO_2_ quenching based sensing platform for OTA molecule detection: (**a**) in the absence of target analyte, adsorption of FAM-labeled aptamer on TiO_2_ surface led fluorescence quenching; and (**b**) in the presence of target analyte, the anti-parallel G-quadruplex structure form decrease adsorption and fluorescence recovered [102].

The fluorescence intensity of dyes can be quenched by gold NPs as a result of fluorescence resonance energy transfer and collision processes between these two [94]. The degree of quenching is dependent on the size and shape of the nanoparticles [95]; for instance, well dispersed nanosize gold NPs significantly quench the intensity, while aggregated particles are not efficiently quenched [94]. Based on aggregation phenomena, various optical and electrochemical aptasensors were developed for molecules that trigger the aggregation by using different strategies. Salt induced aggregation of gold NP and disaggregation based on reversible assembly of ssDNA on the surface of particles which promotes the quenching of the dye, is one of the most popular sensing strategies used in fluorescence based aptasensors [96,97,98,99]. In another study, Emrani et al. [100] developed a fluorescent aptasensor based on hairpin structure of complementary strand of aptamer and used gold NPs as fluorescence quencher, and streptavidin coated silica NPs (SNPs) as amplifier. In the absence of target, fluorophore is in close proximity to the surface of gold NPs, leading a weak fluorescence emission. When target is added to the system, fluorophore comes to close proximity to the surface of SNPs because of the formation of hairpin structure of complementary strand resulting in a very strong fluorescence emission. Several other aptasensors based on quenching effect of carbon nanotubes [101], mesoporous carbon nanospheres [102], titanium dioxide [103] (Figure 2) and graphene oxide [104] were developed. 

## 3. Nano-Aptasensing for Mycotoxins Analysis

Given the numerous advantages of integrating nanomaterials in aptasensing, there has been an increasing interest in developing accurate nanomaterial-assisted aptasensors for mycotoxin analysis in the last years. We discuss in this section the different methods based on the combination of nanomaterials with aptamers described in the literature and applied for mycotoxins monitoring in foodstuffs. General principle will be described with particular emphasis on nanomaterials role in the aptasensing strategy. Moreover, the analytical performances of the different methods will be overviewed, in particular, sensitivity and applicability to real food samples. 

### 3.1. Ochratoxins

The first mycotoxin that has been targeted by aptasensors was ochratoxin A (OTA) which is the most toxic and widespread ochratoxin in food. OTA is a lactone bioproduced by several species of *Aspergillus* and *Penicillium* genera. Given the high toxicity and the widespread of OTA in our food, there have been great advances in OTA detection from traditional chromatographic assays to micro and nanodevices [105,106]. 

**Figure 3:**
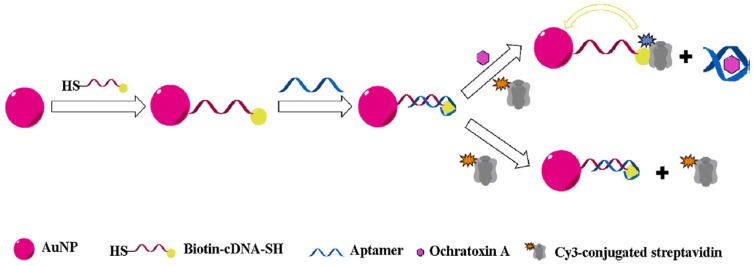
Schematic illustration of the aptasensor based on shielding effect-induced FRET inhibition for OTA detection, reproduced with permission from [55]. Copyright Elsevier, 2016.

Owing to their unique optical and electronic properties, AuNPs are the most extensively used nanomaterials in the design of aptasensors; in particular, electrochemical, fluorescent and colorimetric ones. AuNPs have been used as signal generating probe in colorimetric assays due to their strong vibrant color of the colloidal solution resulting from the surface plasmon resonance (SPR) absorption [107]. We reported in our lab, the first colorimetric OTA aptasensor based on the target-induced color change of AuNPs. In this work, OTA aptamer protects AuNPs against salt-induced aggregation. Upon addition of OTA, the aptamer conformation changes from a random coil structure into a rigid G-quadruplex unable to stabilize AuNPs. This protection/deprotection of AuNPs is associated with a color change easily detected by naked eye. Using this principle, OTA was detected in the range of 8.07–252.38 µg/L with a LOD of 8.07 µg/L [108]. The sensitivity of the proposed assay was not sufficient to meet the maximum allowed limits fixed by the European commission summarized in table. The lack of sensitivity was explained by the number of NPs required to generate a significant color change. A molecular excess of targets is thus necessary to assemble of diassemble the aggregates [109]. Aiming to overcome these limitations, Xiao et al. described a colorimetric aptasensor based on the disassembly of aggregates of oriented AuNP dimers by target molecules. This AuNPs dimer-based sensor has shown better stability, sensitivity (LOD = 0.02 µg/L) and detection dynamic range (0.08–100.8 µg/L). Furthermore, it was noted that the disassembly of AuNPs dimmers was faster than that of large aggregates reducing thus the analysis time [109]. Besides colorimetric assays, gold nanomaterials have also been employed in the development of Localized Surface Plasmon Resonance (LSPR)-based aptasensors. In the assay described by Park et al., the presence of OTA induces the aptamer folding into a G-quadruplex structure resulting in a longitudinal wavelength shift of the LSPR peak associated with a change in the local refractive index near the gold nanorod surface. The proposed label-free assay reached a LOD of 0.4 µg/L with a good applicability on ground corn samples [50]. Moreover, AuNPs are known as fluorescence quenchers for conventional dye donors [110]. Liu et al. described a fluorescent aptasensor for OTA detection by using AuNPs as acceptor and Cy3-streptavidin as FRET (Fluorescence energy transfer) donor. In the absence of OTA, half of the aptamer hybridizes with the biotinylated complementary sequence immobilized on AuNPs, while the other half blocks the biotin–streptavidin interaction, inhibiting the FRET from Cy3-conjugated streptavidin to AuNPs. After OTA binding, the shielding effect-based FRET inhibition is reduced, thus leading to dose-dependent fluorescence decrease. As compared to the assays described above, this aptasensor showed a high sensitivity (LOD = 1.4 ng/L) with a good applicability in spiked wheat and green coffee bean extractives. This may return to the fact that the aptamer was used without any conjugation resulting in a free folding and enhanced affinity to the target [56] (Figure 3). In another report, AuNPs have been used as quencher to silica nanoparticles coated with streptavidin (used as fluorescence enhancers) to construct a fluorescent aptasensor for OTA detection in grape juice and serum samples. No fluorescence emission was observed in the absence of OTA because of the hybridization of AuNPs modified-aptamer with its FAM and biotin-modified complementary sequence (CS). However, upon OTA addition, the aptamer binds to its target and releases the CS allowing its interaction with streptavidin, resulting in a very strong fluorescence emission. This strategy allowed the detection of OTA down to 0.039 µg/L with excellent recoveries in apple juice and serum samples [111]. In other reports, AuNPs have been used as signal amplifiers. As an example, Yang et al. described an electrochemical aptasensor based on two-level cascaded signal amplification strategy through the aptamer-based sandwich model. AuNPs has been used as first level signal enhancer, while a larger number of guanine-rich DNA was bound to the NPs surface to provide abundant anchoring sites for methylene blue to achieve the second-level signal amplification. The proposed method provided an amplified electrochemical response: 8.5 (±0.3) folded signal intensity with the low LOD of 0.0003 µg/L [112]. Similarly, Jiang et al. fabricated a signal amplification platform based on AuNPs-reduced graphene oxide conjugate for the impedimetric detection of OTA in wine. The fabricated platform was characterized by a large surface area providing an excellent carrier for DNA reporters. In this work, a single hybridization event between OTA aptamer and reporter DNA was translated into more than 10^7^ redox events, increasing the charge-transfer resistance (Rct) by 7~ orders of magnitude compared with that of the free aptamer modified electrode [113]. The use of AuNPs as immobilization support has been also reported. Huang et al. immobilized OTA aptamer on AuNPs/molybdenum selenide nanoflowers (MoSe_2_) modified electrode. Then, OTA detection was carried out electrochemically with a low LOD (0.00003 µg/L). This remarkable sensitivity returns to the large surface area of MoSe_2_ and the good conductivity of AuNPs [114]. Aptamer modified AuNPs based strip assay methods were also developed for on-site rapid analysis of OTA with a visual LOD of 1 ng/mL [115,116]. 

Silver nanomaterials have also been also used in OTA aptasensing either as aptamer carriers or as signal generating probes. Evtugyn et al. immobilized OTA aptamer on a gold electrode covered with electropolymerized layers of neutral red and Ag nanoparticles decorated with macrocyclic ligands. In the presence of OTA, the aptamer conformational switch changes the sensing layer properties and increases the charge transfer resistance monitored by electrochemical impedance. The assay allowed the detection of OTA with a LOD of 0.02 µg/L and a good applicability in spiked beer samples. In this work, the incorporation of AgNPs offered a regular composition of the surface layer and greater changes in its permeability for small charge carriers enhancing thus the aptasensor performance [117]. Recently, the use of aptamer functionalized AgNPs probes for OTA detection using nano impact electrochemistry has been described. The proposed method was based on measurements of the individual collision events aptamer-functionalized AgNPs and a carbon fiber microelectrode. OTA binding induced collision frequency changes enabling a single step detection. In contrast to OTA aptasensors described previously, this method is based on a single step procedure employing the electrode and an aptamer-functionalized NPs [118]. In another report, chen et al., employed a DNA-scaffolded-silver-nanocluster (AgNCs) as a fluorophore for the fluorescent detection of OTA. The proposed aptasensor reached a high sensitivity (LOD = 0.002 µg/L). This study provides a promising technique for mycotoxins detection benefiting from the unique fluorescence properties of AgNCs including high quantum yields, high stability, good biocompatibility and nontoxicity [119].

Given the optical quality of quantum dots (QDs); excellent photo-stability, broad excitations, as well as strong and tunable emissions, these nanocrystals have been applied in a wide variety of biosensing schemes in different fields [120]. They have been used as individual signal generating probe or combined to other nanostructures. Wang et al. described the first fluorescent strip based on aptamer-QDs technology for OTA detection in wine samples. OTA monitoring was performed within 10 min with a LOD of 1.9 µg/L [121]. Later on, Hao et al. combined QDs to nitrogen doped graphene (NGQDs) and silica nanoparticles (SiO_2_ NPs) to fabricate a signal indicator for the fluorescent and electrochemiluminescent (ECL) determination of OTA in peanuts. The as prepared nanocomposite was used to tag the cDNA, while OTA aptamer was immobilized on core–shell Fe_3_O_4_@Au magnetic beads. After formation of OTA-aptamer complex, the preloaded NGQDs@SiO_2_ NPs was partially released providing a dual-channel for OTA detection with the LODs of 0.0005 and 0.012 µg/L for ECL and fluorescence, respectively. This strategy may provide a bridge between a highly sensitive ECL assay and a rapid FL assay and can be applied for the monitoring of a variety of toxins [122]. Recently, the FRET mechanism from graphene quantum dots to cerium oxide NPs has been explored for the ratiometric fluorescence aptasensing of OTA. Two probes were designed; DNA1-gQDs and DNA2-nanoceria. In the absence of the aptamer, the two sequences adsorb and induce a FRET mechanism. In presence of OTA-aptamer complex, FRET was interrupted/recovered, where the fluorescence recovery depends on OTA amount in the sample. By comparing the method to OTA aptasensors available in the literature, the authors confirmed that the proposed ratiometric aptasensor is simple to operate with a low background signal exhibiting a high sensitivity (LOD = 0.0025 µg/L) and good selectivity [123]. Similarly, Chu et al. developed another FRET system based on thick shell quantum dot as acceptor and OTA as a donor. In this work, OTA aptamer was modified with phosphorothioate bases to allow its self-assembly on QDs. In the presence of the target, OTA and QDs form a donor-acceptor pair in close enough proximity enabling a FRET process. OTA amount in the sample has been then monitored by measuring the enhancement of fluorescence. A LOD of 0.5 µg/L has been reached and the aptasensors has been applied for OTA detection in beer samples with good recoveries. This study demonstrated that hick–shell QDs provide an ideal alternative for highly sensitive imaging and intensive illumination in the fields of biotechnology and bioengineering [124]. Combined to other nanomaterials, QDs have been also used as signal amplifiers. Hao et al. constructed an aptasensor by combining two nanocomposites of AuNPs functionalized silica-coated iron oxide magnetic nanoparticles (mSiO_2_@Au) and QDs-modified graphene/AuNPs nanocomposites (GAu/CdTe). Using the synthesized nanocomposites, the electrochemical aptasensing of OTA was carried out based on a dual signal amplification strategy with a low LOD (0.00007 µg/L). This promising technique constitutes one of the most sensitive aptasensors for OTA detection [125]. Finally, QDs have been used in fluorescence quenching based aptasensing of OTA. In a recent study, molybdenum disulfide (MoS_2_) nanosheet has been used as quencher to QDs labeling OTA aptamer. In the presence of OTA, the aptamer folding induces an OTA concentration-dependent recovery of the fluorescence intensity. Because of the involving of semiconductor QDs, the proposed system may provide longer fluorescence lifetime and more choices of emission/excitation wavelengths than the reported sensors based on the fluorescence quenching effects of MoS_2_ nanosheets [126].

Other nanomaterials have been used in OTA aptasensing; such as nanoceria for its redox properties [127], single-walled carbon nanotubes as signal quenchers [128] or electrochemical signal amplifiers [129], nanographite as fluorescence quencher [130] and fluorescent nanoparticles. Table 1 provides analytical characteristics of literature reported nanomaterials based aptasensors for the detection of OTA (Table 1). 

### 3.2. Aflatoxins

Aflatoxins are a group of polyketide produced by many species of *Aspergillus* genus. In contrast to OTA, few papers studied aflatoxin aptasensing based on nanomaterials, where the major part of the reported aptasensors used metallic nanoparticles, mainly gold and silver. Luan et al. employed AuNPs as a colorimetric probe for the detection of AFB_1_ and AFB_2_. The reported assays were simply based on the target induced aggregation of gold nanoparticles. The color change was detectable with bare eye and microplate readout. The first assay allowed the detection of AFB_1_ with a LOD of 0.025 µg/L, while AFB_2_ aptasensor reached a detection limit of 0.025 µg/L with a good applicability in beer samples [132,133]. In another report, Hosseini et al. developed a colorimetric assay based on the same principle of target induced AuNPs aggregation. In the same report, the authors investigated the catalytic activity of the aggregated nanoparticles which increased the chemiluminescence reaction in the presence of luminol and hydrogen peroxide. A better sensitivity has been noted by using the chemiluminescence detection; the LOD decreased from 7 nM to 0.5 nM [134]. Florescence recovery based aptasensors have been also reported for aflatoxins determination in food. Wang et al. used AuNPs as quenching element of the fluorescent nitrogen-doped carbon dots (N,C-dots) assembled on AuNPs-modified aptamer. In the presence of AFB_1_, the fluorescence of the N,C-dots recovered and the measurement of Fl intensity allowed the detection of AFB_1_ with a LOD of 0.005 µg/L. The assay applicability has been demonstrated on in real corn samples with a high sensitivity better than that obtained with HPLC [135]. For amplified detection of AFB_1_, Zheng et al. developed an electrochemical aptasensor based on a dual enzymatic amplification strategy using telomerase and EXO III. In this work, AuNPs have been used as carrier of the complementary DNA of AFB_1_ aptamer. Taking advantage of this two-round signal amplification strategy, both the sensing range and LOD of were greatly improved by a three-order magnitude widening and about 1000-fold enhancemen, respectively, with the low LOD of 6 × 10^−11^ µg/L [136]. Gold nanomaterials have been also employed as SERS signal enhancer combined to silver NPs. Zhao et al. fabricated two kinds of SERS labels embedded solid Ag core and Au Schell NPs which provide a strong plasmonic coupling in addition to a great amplification of the SERS signal of Raman labels. Then, a SERS engineered Raman aptasensors were developed for the double detection of the simultaneous detection of OTA AFB in the maize meal. This was the first sensitive SERS signal dependent allowing double detection of mycotoxins with the respective LODs; 0.006 and 0.03 µg/L [137]. In a recent study, a novel SERS based aptasensor has been developed for AFB_1_ detection. In this work, the aptamer conjugated magnetic-beads and the gold nanotriangles (GNTs)-DTNB@Ag-DTNB nanotriangles were used as the capturer and the reporter of AFB_1_, respectively. In the absence of AFB1, the reporter nanoprobes did not assemble with the capture ones and no Raman signal was recorded. After addition of AFB_1_, an assembly of the reporter nanoprobes, AFB_1_ and capture nanoprobes was noted resulting in a high Raman signal allowing the the quantitative trace detection of AFB_1_ in peanut oil. The proposed assay reached a high sensitivity (0.00054 µg/L) owing to the strong Raman enhancement effect of GNTs [138]. Finally, DNA scaffolded silver nanoclusters have been also employed as signal enhancers in the fluorescent aptasensing of AFB_1_. The platform has been applied for the simultaneous detection of OTA and AFB_1_ by immobilizing the corresponding aptamers on magnetic beads hybridizing with signal stranded signal probes 1 and 2. In the presence of the targets, the signal probes are dissociated from aptamers and acted as the corresponding scaffolds to synthesize AgNCs with different photoluminescence emission bands. Using this system, OTA and AFB_1_ have been detected with the respective LODs of 0.0002 and 0.0003 µg/L [139].

**Figure 4:**
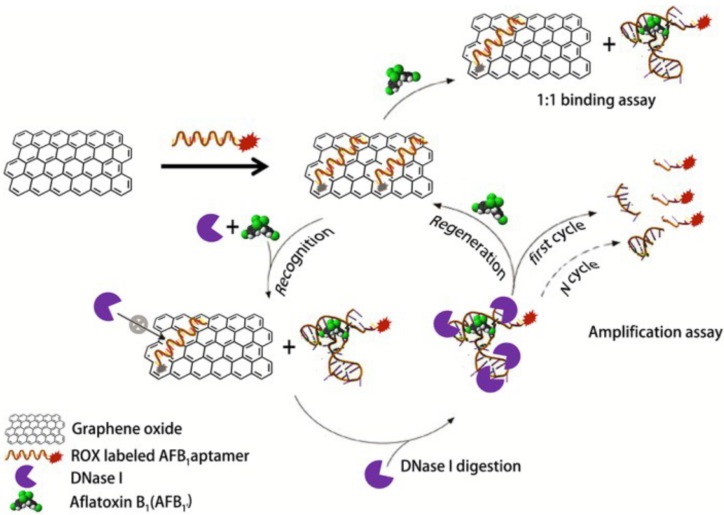
Fluorescent Assay methods for the detection of AFB_1_ based on DNA aptamer and GO, reproduced with permission from [136]. Copyright Royal Society of Chemistry, 2010.

In other reports, Graphene oxide nanomaterials have been combined to aptamers to construct accurate aptasensors for AFB_1_ detection. First, a fluorescence recovery based assay has been developed. In this work, GO played the role of a quencher of the fluorescent quantum dots labeling AFB_1_ aptamer. The assay exhibited a LOD of 0.31 µg/L and a good applicability in peanut oil samples [140]. Later on, Zhang et al. developed a fluorescent sensor based on the ability of graphene oxide to protect aptamers from nuclease cleavage for amplified detection and nanometer size effect of GO to tune the dynamic range and sensitivity. As shown in Figure 4, the detection was simple and rapid, it can be realized by incubating the contaminated sample with the aptamer, nuclease and graphene oxide [141] (Figure 4). There is also report on the development of dipstick assay for the detection of AFB_1_ [142] which can take advantages of nanomaterials to design future rapid and robust assays for mycotoxins monitoring. Nanomaterial-assisted aptasensors for aflatoxins, zearalenone and fumonisin B1 determination are summarized in Table 2 (Table 2).

### 3.3. Other Mycotoxins

After OTA and aflatoxins, the aptamers recognizing fumonisin B1 and zearalenone have been selected [146,147]. There are few reports in the literature describing nanomaterial-assisted aptasensors for ZEN mycotoxin. Wu et al. explored the fluorescence of up conversion NPs to develop a highly sensitive ZEN aptasensor. For that, ZEN aptamer has been immobilized on magnetic nanoparticles and used as capture probe, whereas the complementary strand has been labeled with UPCNPs and used as signal probe. In the presence of ZEN, the aptamer binds to its target and releases the cDNA resulting in a decrease of the fluorescence intensity. The monitoring of luminescence allowed the quantitative detection of ZEN with a low limit of detection (0.007 µg/L). In addition to the high sensitivity, excellent reorganization, specificity and wide linear range have been achieved using the developed system [143].

**Figure 5:**
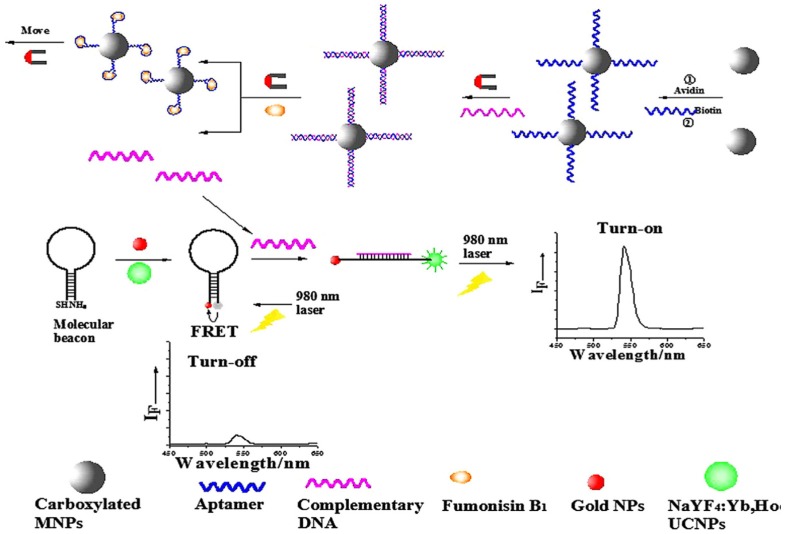
Schematic illustration of the fluorescence resonance energy transfer between NaYF4: Yb, Ho UCNPs and AuNPs based on molecular beacons for fumonisin B 1 sensing, reproduced with permission from [139]. Copyright Elsevier, 2013.

Fumonisin B_1_ (FB_1_) is a mycotoxin produced by the fungus *Fusarium verticillioides*, which commonly infects corn and other agricultural products. FB_1_ is neurotoxic, hepatotoxic, and nephrotoxic in animals, and it has been classified as a possible carcinogen to humans [148]. By using FB_1_ aptamer, Wu et al. developed the first FRET aptasensor based on up conversion nanoparticles and AuNPs. The aptasensing strategy was based on a molecular beacon modified with the quencher (AuNPs) at 5′ and with the donor (UPCNPs) at 3′. In the presence of FB_1_, the molecular beacon undergoes a spontaneous conformational change, which causes the UCNPs and AuNPs to detach from each other, leading to a fluorescence recovery (Figure 5). This phenomenon allowed FB_1_ monitoring with a LOD of 0.01 µg/L. For real sample application, the method was used successfully to monitor FB_1_ levels in naturally contaminated maize samples [145]. Recently, we developed in our lab an aptamer-based fluorescence quenching assay for ZEN detection. In this work, functional graphene oxide has been used as quencher of the fluorescently labeled aptamer. However, after ZEN addition, a fluorescence recovery proportional to the target concentration has been noted. Using this principle, ZEN has been detected with a LOD of 0.6 µg/µL. Moreover, the authors noted that the synthesized graphene oxide exhibited efficient quenching property and very good dispersibility in water, which are essential for applications to analytes of real-world food samples [144].

## 4. Conclusions and Prospects

Nanomaterials owing to their unique physiochemical characteristics can provide diverse ways to design a variety of aptasensors. Unlike antibodies, aptamers undergo structure switchable conformational changes upon target analyte binding, making them a promising bioreceptor to explore all possible features of nanomaterials. Owing to their nanoscale size and potential to work reversibly, structure-switchable nano aptamer-based assays are very well adopted for the continuous and real time monitoring of mycotoxins in very complex environments. In this direction, a large number of novel and new nanomaterials have been explored to perform various functionalities in aptasensing platform. However, the current state of the art of aptamer-conjugated nanomaterials is yet to improve due to difficulties in bioconjugation chemistry, and the lack of intrinsic properties and functional moieties in some of the nanomaterials. To overcome these challenges, researchers are rapidly improving the existing procedures, maximizing the advantages of nanomaterials. For example, research in this direction is focused on the synthesis of composite/hybrid materials, quantum structures and functionalized nanomaterials. While talking about the mycotoxins, although a variety of nanomaterials have been employed as immobilization support, but other functionalities such as single generating probe, single amplification are not fully explored in the construction of aptasensors. Gold and silver nanoparticles are the mainly employed materials, especially for optical output in the construct of aptasensor. However, properties of both these materials are highly dependent on pH, temperature, nature of the medium and size of the materials, and are prone to a large extent variation under different physiological environment. On the other hand, aptamer-based assays for mycotoxins apart from OTA are still in development phase as compared to immunoassays. Although aptamers, for OTA and AFB_1_, are well established in the literature, they are very recently developed or yet to develop for other mycotoxins. Moreover, despite offering the advantages over other bioreceptors, aptamers still face challenges that have hampered the commercialization of aptamer based assays. A major drawback is the difficulties designing aptamers for small size molecule and subsequent determination of Kd for such aptamers. Because of this problem associated with Kd measurement, several complementary methods may be required to improve the binding affinity of aptamers with their targets. 

It is anticipated that the future research may focus on overcoming the hurdles in the direction of commercialization of aptamer based assays. For example, material scientists can work to explore new functional nanomaterials by employing different synthetic procedures, which can be further employed in the fabrication of aptasensors with improved analytical figures of merit. Moreover, particularly for mycotoxin analysis, OTA nanomaterial reported strategies to design aptamer-based assays can be extended for the analysis of other mycotoxins such as fumonisin B1, aflatoxin B1 and zeeralenone. 

## Figures and Tables

**Table 1:** Nanomaterial-assisted aptasensors for OTA determination.

Nanomaterial	Nanomaterial Role	Limit of Detection (µg/L)	Real Sample	Refs.
AuNPs	Colorimetric probe	8.07	------	[108]
	Colorimetric probe	0.02	Red wine	[109]
	LSPR probe	0.4	Ground corn samples	[50]
	FRET quencher	1.4 × 10^−3^	Wheat and green coffee beans	[56]
AuNPs and silica NPs	Fluorescence quencher	0.039	Grape juice and serum	[111]
	Signal amplifier	0.0003	Red wine	[112]
AuNPs-rGo	Signal amplifier	0.0003	Red wine	[113]
AuNPs-MoS_2_	Immobilization	0.00003	Wine	[114]
AgNPs	Immobilization	0.02	Beer	[117]
AgNPs	Signal generating probe	0.02	-----	[118]
AgNCs	Fluorophore	0.002	Wheat	[119]
QDs	Fluorescent probe	1.9	Wine	[121]
NGQDs@SiO_2_ NPs	ECL and Fluorescent probe	0.0005 0.012	Peanut	[122]
Graphene quantum dots and nanoceria	FRET probe	0.0025	Peanut	[123]
Thick shell QDs	FERT probe	0.5	Beer	[124]
mSiO_2_@Au QDs-modified graphene/AuNPs	Signal amplifier	0.00007	------	[125]
QDs and MoS_2_	Fluorescent probe and quencher	1	Red wine	[126]
Nanoceria	Redox probe	0.06	Milk	[127]
SWCNTs	Fluorescence quencher	9.72	Beer	[128]
SWCNTs	Signal amplifier	0.02	Serum and grape juice	[129]
Nanographite	Fluorescence quencher	8.07	Red wine	[130]
Fluorescent NPs	Fluorescence probe	0.002	Beer	[131]

**Table 2:** Nanomaterial-assisted aptasensors for aflatoxins, zearalenone and fumonisin B1 determination.

Aflatoxin	Nanomaterial	Role of the Nanomaterial	LOD (µg/L)	Real Sample	References
AFB_1_	AuNPs	Colorimetric probe	0.025	------	[132]
AFB_2_		Colorimetric probe	0.025	Beer	[133]
AFB1		Colorimetric probe	2.18	Peanut and rice	[134]
		HRP-mimicking activity	0.15	Peanut and rice	[134]
		Fluorescence quencher	0.005	Corn	[135]
		cDNA carrier	6 × 10^−11^	Corn	[136]
	Ag core and Au shell NPs	SERS signal enhancer	0.03	Maize	[137]
	GNTs/Ag core–shell	SERS signal enhancer	0.00054	Peanut oil	[138]
	AgNCs	Fluorescent signal enhancer	0.0003	Rice, corn and wheat	[139]
	Graphene oxide NPs	Signal amplification	0.35	Corn	[141]
	QDs/Graphene oxide NPs	Fluorescence probe/Quencher	0.31	Peanut oil	[140]
ZEN	UPCNPs	Florescent probe	0.007	Beer	[143]
	Functional graphene oxide	Fluorescence quencher	0.5	Wine and beer	[144]
FB1	UPCNPs and AuNPs	FRET probe	0.01	Maize	[145]
